# Supplementary material for: Assessment of current good manufacturing practice (cGMP) compliance in pharmaceutical manufacturers in Ethiopia: Cross-sectional descriptive study
Source: PLoS One. 2026 Mar 9;21(3):e0343881. doi: 10.1371/journal.pone.0343881 (PMC12970858; doi:10.1371/journal.pone.0343881)
Supplement: S1 Table — (DOCX) [file pone.0343881.s001.docx]

| S1 Table. Supplementary file; General Overview of the GMP Sub-Elements Compliance Scores of Pharmaceutical Manufacturers in Ethiopia, 2024(n = 6) | | | | | | | |
| --- | --- | --- | --- | --- | --- | --- | --- |
| **Items** | **Status** | Number and percentage of rating scores for each company (Companies 1 through 6) | | | | | |
|  |  | 1 | 2 | 3 | 4 | 5 | 6 |
| Quality Assurance: Total assessed items #9 | Not compliant | - | - | - | - | - | - |
|  | Partially compliant | 5(55) | - | - | - | - | - |
|  | Fully compliant | 4(45) | 9(100) | 9(100) | 9(100) | 9(100) | 9(100) |
| QRM system:  Total assessed items: #7 | Not compliant | 1(14.3) | - | - | - | - | - |
|  | Partially compliant | 6(85.7) | 1(14.3) | 1(14.3) | 1(14.3) | 5(71.4) | 5(71.4) |
|  | Fully compliant |  | 6(85.7) | 6(85.7) | 6(85.7) | 2(28.6) | 2(28.6) |
| Premise and facility:  Total assessed items: #10 | Not compliant | 2(20) | - | - | - | - | - |
|  | Partially compliant | 4(40) | 1(10) | 1(10) | 1(10) | 1(10) | 1(10) |
|  | Fully compliant | 4(40) | 9(90) | 9(90) | 9(90) | 9(90) | 9(90) |
| Personnel:  Total assessed items: #6 | Not compliant | - | - | - | - | - | - |
|  | Partially compliant | 2(33.3) | 1(16.7) | 1(16.7) | 1(16.7) | 1(16.7) | 4(66.7) |
|  | Fully compliant | 4(66.7) | 5(83.3) | 5(83.3) | 5(83.3) | 5(83.3) | 2(33.3) |
| Hygiene and sanitation:  Total assessed items: #9 | Not compliant | - | - | - | - | - | - |
|  | Partially compliant | 2(22.2) | - | - | 2(22.2) | - | - |
|  | Fully compliant | 7(77.8) | 9(100) | 9(100) | 7(77.8) | 9(100) | 9(100) |
| Validation system:  Total assessed items: #8 | Not compliant | - | - | - | - | - | - |
|  | Partially compliant | - | - | 1(12.5) | 1(12.5) | - | - |
|  | Fully compliant | 8(100) | 8(100) | 7(87.5) | 7(87.5 | 8(100) | 8(100) |
| Documentation  Total assessed items: #13 | Not compliant |  | - | - | - | - | - |
|  | Partially compliant | 2(15.4) |  |  | 4(30.8) |  |  |
|  | Fully compliant | 11(84.6) | 13(100) | 13(100) | 9(69.2) | 13(100) | 13(100) |
| HVAC system:  Total assessed items: #10 | Not compliant | - | - | - | - | - | - |
|  | Partially compliant | 3(30) | 1(10) | - | 1(10) | - | - |
|  | Fully compliant | 7(70) | 9(90) | 10(100) | 9(90) | 10(100) | 10(100) |
| Equipment: Total assessed items: #8 | Not compliant | - | - | - |  | - | - |
|  | Partially compliant | 6(75) | 2(25) | 2(25) | 1(12.5) | - | 1(12.5) |
|  | Fully compliant | 2(25) | 6(75) | 6(75) | 6(75) | 8(100) | 7(87.5) |
| Materials: Total assessed items: #13 | Not compliant | - | - | - | - | - | - |
|  | Partially compliant | 1(7.7) | 2(15.4) | 2(15.4) | - | - | - |
|  | Fully compliant | 12(92.3) | 11(84.6) | 11(84.6) | 13(100) | 13(100) | 13(100) |
| Production process: Total assessed items: #8 | Not compliant | - | - | - | - | - | - |
|  | Partially compliant | 1(12.5) | 1(12.5) | 1(12.5) | 1(12.5) | 1(12.5) | 1(12.5) |
|  | Fully compliant | 7(87.5) | 7(87.5) | 7(87.5) | 7(87.5) | 7(87.5) | 7(87.5) |
| Quality control  : Total assessed  items: #11 | Not compliant | - | - | 1(9.1) | - | - | - |
|  | Partially compliant | 1(9.1) | 1(9.1) | - | - | 1(9.1) | - |
|  | Fully compliant | 10(90.9) | 10(90.9) | 10(90.9) | 11(100) | 10(90.9) | 11(100) |
| Regulatory compliance: Total assessed  items: #2 | Not compliant | - | - | - | - | 1(50) | - |
|  | Partially compliant | 2(100) | - | - | - | - | - |
|  | Fully compliant | - | 2(100) | 2(100) | 2(100) | 1(50) | 2(100) |
| Internal audit: Total assessed  items: #4 | Not compliant | - | 1(25) | - | 1(25) | 1(25) | - |
|  | Partially compliant | 4(100) | 3(75) | 4(100) | 3(75) | 3(75) | 4(100) |
|  | Fully compliant | - | - | - | - | - | - |
| Compliant and recall:  Total assessed  items: #4 | Not compliant | - | - | 1(25) | - | 1(25) | - |
|  | Partially compliant | 1(25) | 1(25) | - | - | 1(25) | 2(50) |
|  | Fully compliant | 3(75) | 3(75) | 3(75) | 4(100) | 2(50) | 2(50) |
| Supplier management: Total assessed  items: #4 | Not compliant | - | - | - | - | - | - |
|  | Partially compliant | 2(50) | - | 4(100) | 2(50) | - | - |
|  | Fully compliant | 2(50) | 4(100) | - | 2(50) | 4(100) | 4(100) |

*Source: Checklist Survey on GMP Compliance in Ethiopian Pharmaceutical Companies, July 10 to September 16, 2024*.
