# Supplementary material for: Assessment of current good manufacturing practice (cGMP) compliance in pharmaceutical manufacturers in Ethiopia: Cross-sectional descriptive study
Source: PLoS One. 2026 Mar 9;21(3):e0343881. doi: 10.1371/journal.pone.0343881 (PMC12970858; doi:10.1371/journal.pone.0343881)
Supplement: S1 Checklist — (DOCX) [file pone.0343881.s002.docx]

**Annex I: - Information Sheet and Consent Form**

**Respondent information sheet**

- Title of the research: - “Assessment of risk management system in the pharmaceutical industries in Ethiopia
- Name of Principal Investigators: Teka Benti, Dr Dereje Kebebe
- Organization/university: Jimma University, Wollega University
- Benefit of the study: It can benefit your company by identifying areas that require immediate attention to prevent further risks and solve existing problems.
- Procedures: Documentation review, observation, and information from concerned personnel via self-administered questionnaires are methods for data collection.
- Risk and Discomforts: Participating in this study will not affect you or your company's reputation.
- Benefits: It is important to review the activities of the company to identify its own strengths and weaknesses.
- Confidentiality: De-identifying study data by using a code number instead of the participant's real name, and ensuring that the data will be handled securely
- Right to refuse or withdraw: Your participation in this study is voluntary.

**Contact information**:

Principal investigator: Teka Benti, Email: tekabenti55@gmail.com

Dereje Kebebe (Ph.D., Associated professor of pharmaceutics) Email: [dereje.kebebe@ju.edu.et](mailto:dereje.kebebe@ju.edu.et)

Desta Asefa (MSc, Assistant professor of pharmaceutics) Email: [desta4best@gmail.com](mailto:desta4best@gmail.com)

**Consent Form**

I am informed fully in the language I understand about the aim of above-mentioned research. I understood the purpose of the study entitled “*Assessment of risk management system in the pharmaceutical industries in Ethiopia*”. I have been informed this study which involves collecting ___________sample. I have also read the information sheet or it has been read to me. In addition, I have been told all the information collected throughout the research process will be kept confidential. I understood my current and future right will not be affected if I refused to participate or with draw from the study. I ____________________, after being fully informed about the detail of this study, hereby gave my consent to participate in this study and approve my agreement with signature.

Employee signature ___________ Date___________/____________/_______________

Investigator signature ___________Date__________/___________/_________________

Thank you

**Annex: A. Checklist**

**GMP Checklist for Domestic Pharmaceutical Companies**

Code assigned to the company________________

Factory-responsible confirmation: where ‘1’ referred to non-compliance, ‘2’,

Partial compliance and ‘3’, full compliance. Please fill the box with appropriate one accordingly

| **SN** | **GMP Elements** | **Sub-element** | | | Rating | | |
| --- | --- | --- | --- | --- | --- | --- | --- |
|  |  |  |  |  | 1 | 2 | 3 |
| 1 | QA system | 1 | Provision of specialized QA department and responsibility | |  |  |  |
|  |  | 2 | QA activities documentation | |  |  |  |
|  |  | 3 | Design and development of pharmaceutical products considering GMP | |  |  |  |
|  |  | 4 | Specification of production and control operations in written form | |  |  |  |
|  |  | 5 | Clearly defined managerial responsibilities | |  |  |  |
|  |  | 6 | Arrangements for correct starting and packaging materials | |  |  |  |
|  |  | 7 | Controls on starting materials, intermediate products, bulk products, in-process controls, calibrations, and validations | |  |  |  |
|  |  | 8 | Storage, distribution, and handling of pharmaceutical products | |  |  |  |
|  |  | 9 | Procedure for self-inspection and/or quality audit | |  |  |  |
| 2 | QRM | 1 | Documentation and regular review of risk assessments | |  |  |  |
|  |  | 2 | Risk evaluation based on scientific knowledge and process experience | |  |  |  |
|  |  | 3 | Establishment of key performance indicators (KPIs) for QRM effectiveness | |  |  |  |
|  |  | 4 | Clearly defined and accessible QRM procedures and documentation | |  |  |  |
|  |  | 5 | Standardized risk assessment methodologies | |  |  |  |
|  |  | 6 | Documentation of regulatory expectations for QRM | |  |  |  |
|  |  | 7 | System for approving changes impacting product quality | |  |  |  |
| 3 | Premise and Facilities | 1 | Design to prevent contamination and cross-contamination | |  |  |  |
|  |  | 2 | Adequate space for equipment and materials placement | |  |  |  |
|  |  | 3 | Regular cleaning and maintenance of facilities | |  |  |  |
|  |  | 4 | Documented cleaning schedule and procedure | |  |  |  |
|  |  | 5 | Logical layout of premises for production | |  |  |  |
|  |  | 6 | Maintenance to avoid quality hazards | |  |  |  |
|  |  | 7 | Waste management provisions | |  |  |  |
|  |  | 8 | Construction with smooth and easy-to-clean surfaces | |  |  |  |
|  |  | 9 | Prevention of unauthorized entry | |  |  |  |
|  |  | 10 | Ancillary areas facility | |  |  |  |
| 4 | Personnel | 1 | Sufficient qualified personnel | |  |  |  |
|  |  | 2 | Organization Chart | |  |  |  |
|  |  | 3 | Qualified, trained, and experienced staff | |  |  |  |
|  |  | 4 | On-the-job training program | |  |  |  |
|  |  | 5 | Certification and documentation of specialized training | |  |  |  |
|  |  | 6 | Specific training for personnel in contamination hazard areas | |  |  |  |
| 5 | Hygiene & Sanitation | 1 | Staff rest and hygiene facilities | |  |  |  |
|  |  | 2 | Hygiene procedures for production, QC, and storage areas | |  |  |  |
|  |  | 3 | Staff health check-up system | |  |  |  |
|  |  | 4 | Periodic eye examinations for visual inspection staff | |  |  |  |
|  |  | 5 | Training on GMP and hygiene instructions for all personnel | |  |  |  |
| 6 | Validation and qualification | 1 | Proper documentation of validation | |  |  |  |
|  |  | 2 | Conducting validation studies as per protocols | |  |  |  |
|  |  | 3 | Validation of production procedures | |  |  |  |
|  |  | 4 | Formulation procedures for active ingredients | |  |  |  |
|  |  | 5 | Dual-signature weighing and measuring records | |  |  |  |
|  |  | 6 | Validation of equipment and materials | |  |  |  |
|  |  | 7 | Suitability demonstration for new master formula or methods | |  |  |  |
| 7 | Documentation | I | Document control system for GMP-related documents | |  |  |  |
|  |  | 2 | Accuracy and clarity of documentation | |  |  |  |
|  |  | 3 | Retention of records as per regulatory requirements | |  |  |  |
|  |  | 4 | Storage of records to prevent loss or damage | |  |  |  |
|  |  | 5 | Labeling of containers, equipment, or premises | |  |  |  |
|  |  | 6 | Use of status-indicating labels | |  |  |  |
|  |  | 7 | Availability and compliance of specifications, testing procedures, master formulae, packing instructions, and SOPs | |  |  |  |
| 8 | Materials | 1 | Proper storage of materials | |  |  |  |
|  |  | 2 | Testing and approval of raw materials before use | |  |  |  |
|  |  | 3 | Documentation of raw material quality testing | |  |  |  |
|  |  | 4 | Preventing direct contact of non-production materials with products | |  |  |  |
|  |  | 5 | Quarantine of incoming materials and finished products | |  |  |  |
|  |  | 6 | Quality control department approval of starting materials | |  |  |  |
|  |  | 7 | Handling and control of primary and printed packaging materials | |  |  |  |
|  |  | 8 | Designated persons dispensing starting materials | |  |  |  |
|  |  | 9 | Suitability of water used in manufacturing | |  |  |  |
|  |  | 10 | Separation and marking of rejected materials and products | |  |  |  |
|  |  | 11 | Handling of intermediate and bulk products as starting materials | |  |  |  |
|  |  | 12 | Reworking or recovery of rejected products is permitted if quality of final product is not affected | |  |  |  |
|  |  | 13 | Quarantine of finished products until release | |  |  |  |
| 9 | HVAC system | 1 | Environmental monitoring program | |  |  |  |
|  |  | 2 | Adequate air exchange rates in HVAC systems | |  |  |  |
|  |  | 3 | Monitoring and control of environmental conditions | |  |  |  |
|  |  | 4 | Controlled air filtration | |  |  |  |
|  |  | 5 | Determination of air cleanliness levels based on product and process | |  |  |  |
|  |  | 6 | Controlled pressure differentials in HVAC systems | |  |  |  |
|  |  | 7 | HVAC system contribution to personnel comfort | |  |  |  |
|  |  | 8 | Documentation of HVAC and equipment qualification | |  |  |  |
| 10 | Equipment | 1 | Correct installation and qualification of equipment | |  |  |  |
|  |  | 2 | Controls for maintaining required parameters | |  |  |  |
|  |  | 3 | Regular calibration of instruments and equipment | |  |  |  |
|  |  | 4 | Equipment location suitability for operations | |  |  |  |
|  |  | 5 | Design and construction of equipment for operations | |  |  |  |
|  |  | 6 | Removal of defective equipment | |  |  |  |
|  |  | 7 | Scheduled thorough cleaning of equipment | |  |  |  |
|  |  | 8 | Preventive maintenance program and calibration schedule | |  |  |  |
| 11 | Production Processes | 1 | There are written SOPs for all production processes. | |  |  |  |
|  |  | 2 | SOPs followed and deviations are documented and investigated. | |  |  |  |
|  |  | 3 | In process sample testing practice (Process Analytical Technology (PAT)) | |  |  |  |
|  |  | 4 | Batch production records are maintained for each batch. | |  |  |  |
|  |  | 5 | Batch records include complete information, such as quantities of materials used, process parameters, and results of in-process tests. | |  |  |  |
|  |  | 6 | Flow control of materials and staff to avoid contamination and cross-contamination | |  |  |  |
|  |  | 7 | Master production instructions/, master formula card is available. | |  |  |  |
|  |  | 8 | Dedicated tools for maintenance for sensitizing products | |  |  |  |
| 12 | QC lab | 1 | Independence of QC department | |  |  |  |
|  |  | 2 | Quality control system for sampling and testing | |  |  |  |
|  |  | 3 | Appropriate sample retention procedure | |  |  |  |
|  |  | 4 | Retention of sufficient reference samples | |  |  |  |
|  |  | 5 | SOPs for QC lab functional areas | |  |  |  |
|  |  | 6 | Documentation of stability tests | |  |  |  |
|  |  | 7 | Final product review and approval | |  |  |  |
|  |  | 8 | Approved batch release file | |  |  |  |
|  |  | 9 | Adequate facilities and equipment for testing | |  |  |  |
|  |  | 10 | Validation/Qualification system | |  |  |  |
|  |  | 11 | Efficient cleaning service | |  |  |  |
| 13 | Regulatory compliance | 1 | Compliance with regulatory requirements | |  |  |  |
|  |  | 2 | Procedures for ensuring compliance with regulatory changes | |  |  |  |
| 14 | Internal Audits | 1 | Regular internal audits for GMP compliance | |  |  |  |
|  |  | 2 | Documentation and implementation of audit findings | |  |  |  |
|  |  | 3 | CAPA system for addressing deviations and non-conformances | |  |  |  |
|  |  | 4 | Tracking and verification of CAPA actions | |  |  |  |
| 15 | Product compliant and Recall | 1 | Designated person for complaints and recall | |  |  |  |
|  |  | 2 | Prompt initiation of recall operations | |  |  |  |
|  |  | 3 | Recording of decisions and measures taken due to complaints | |  |  |  |
|  |  | 4 | Batch recall and waste destruction records | |  |  |  |
| 16 | Supplier Management | 1 | Qualification and regular assessment of raw material and service suppliers | |  |  |  |
|  |  | 2 | Documentation of supplier audits and performance reviews | |  |  |  |
| **Part II** | | | | | | | |
| 1 | Employee workload | | | Low workload [ ]  medium workload [ ]  high workload [ ] | | | |
| 2 | Employee Training | | | Partially trained [ ]  Fully trained [ ] | | | |
| 3 | Production line suitability | | | Not suitable [ ]  Suitable [ ] | | | |
| 4 | PAT implementation | | | Not implemented well [ ]  Implemented well [ ] | | | |
| 5 | Government support | | | Not supported [ ]  Supported [ ] | | | |
| 6 | GMP certificate | | | Not certified [ ]  Certified [ ] | | | |

**B. Self-administered questionnaire**

**Part I. Demographic**

1. Your departments_______________________

2. Your roles in department:

General Manager [ ] Production Manager [ ] Quality assurance Manager [ ]

R&D manager [ ] Quality control Manager [ ] other (please specify) ____________

3. Your Level of Education:

MSc in Pharmacy related fields [ ] B pharm, BSc in Pharmacy [ ]

MSc in chemistry related fields [ ] BSc. in Biology, chemistry [ ] Other_________

4. Your Work Experience

- 1. years [ ] 6-10 years [ ] >10 years [ ]

**Part II. Open ended questions**

1. Employee Training level for your organizations

2. Production line and its suitability in your company

3. Government support for this company

4. Certificate status of this organization

5. Workload exitance in your company

6. PAT implementation system

**Thank you for your cooperation! It is Greatly Appreciated!**
