# Supplementary material for: Assessment of current good manufacturing practice (cGMP) compliance in pharmaceutical manufacturers in Ethiopia: Cross-sectional descriptive study
Source: PLoS One. 2026 Mar 9;21(3):e0343881. doi: 10.1371/journal.pone.0343881 (PMC12970858; doi:10.1371/journal.pone.0343881)
Supplement: Attachment — Submitted filename: Response to reviewers.docx [file pone.0343881.s005.docx]

**Assessment of current good manufacturing practice (cGMP) compliance in pharmaceutical manufacturers in Ethiopia**

**Regarding the reviewer's comments, I have corrected the manuscript as suggested. The following information should provide clarification to the reviewer.**

The reference issue was fixed by replacing the original reference with the one that contains the information cited in that paragraph.

This study used a WHO-based checklist because it align with national GMP guideline and scored sub-elements (FC=3, PC=2, NC=1) to produce company compliance percentages and identify weak areas (QA, QRM, HVAC, documentation, internal audit, complaint/recall) and raw checklists are uploaded with the file name 's3’.

The numerical conversion (FC=3, PC=2, NC=1) does not mean that the difference between FC and PC is truly the same as the difference between PC and NC. This numerical assignment serves as a code for NC, PC, and FC to categorize each statement on the checklists into non-compliant, partially compliant, and fully compliant, respectively. The final percentage is calculated based on the total count of statements or checklists categorized as above.

The adjusted CGMP compliance: cGMP compliance rating score: 85% - 100% = high, (70% - 84%)= medium, and <70% = low compliance

The risk associated with deficiencies identified during an inspection was the basis for classifying them consistently into Non-Compliant (NC), Partially Compliant (PC), or Fully Compliant (FC) by the two pharmacist assessors, who used the GMP compliance criteria/guidelines.

The assignment of High, Medium, and Low GMP compliance in the pharmaceutical industry is typically performed through a risk-based scoring system applied during internal or regulatory audits/inspections. It is a key part of Quality Risk Management (QRM) as outlined by international guidelines like ICH Q9

**Sample size and selection**: The restriction to large-scale, high-volume pharmaceutical manufacturers in this study, due to time and budget constraints, limits the generalizability of the findings. This is further addressed in the Limitations section.

Five key personnel were selected from each company: those who deeply understood GMP and GMP-related activities, and upon whom the quality of the prepared pharmaceutical products relied. Additionally, there was no difference in ideas among those five individuals during the interview for each company.
