# Supplementary material for: Assessment of current good manufacturing practice (cGMP) compliance in pharmaceutical manufacturers in Ethiopia: Cross-sectional descriptive study
Source: PLoS One. 2026 Mar 9;21(3):e0343881. doi: 10.1371/journal.pone.0343881 (PMC12970858; doi:10.1371/journal.pone.0343881)
Supplement: Attachment — Submitted filename: letter to reviewers.docx [file pone.0343881.s006.docx]

Dear reviewers, our responses to your comments are as follows:

1. **The manuscript's purpose is unclear; clarify why readers need to know about cGMP compliance in Ethiopia.**

*Understanding cGMP compliance in Ethiopia is critical because it serves as the bridge between the country's ambitious public health goals and its industrial economic strategy. As Ethiopia strives to reduce its 80–85% reliance on imported medicines, cGMP serves as the mandatory legal framework to ensure that locally manufactured drugs are safe, effective, and free from contamination. Furthermore, achieving these international standards is the only way for Ethiopian pharmaceutical firms to secure WHO Prequalification, allowing them to compete in global export markets and build a resilient, self-sufficient healthcare supply chain. Dear reviewers, these concepts are found in final paragraph of introduction, the first three lines.*

**2. Does cGMP non-compliance affect international medicine exports from Ethiopia? Is there a broader impact?**

*In short, cGMP non-compliance acts as a glass ceiling for Ethiopia’s pharmaceutical industry, making international exports virtually impossible since most global markets and procurement agencies, mandate WHO-level certification. Beyond lost revenue, the broader impact is a deepened dependency on expensive imports that drain foreign currency and a heightened risk to public health, as substandard manufacturing can lead to ineffective treatments or the dangerous rise of antimicrobial resistance. Ultimately, failing to meet these standards keeps the local industry in a cycle of "folk-standard" production, preventing Ethiopia from becoming a regional drug-discovery hub and contributing to a brain drain of its most talented scientific professionals. Dear reviewers, these concepts are found in final paragraph of introduction, 3^rd^ line.*

**3. Identify the Ethiopian regulatory authority responsible for enforcing WHO GMP compliance.**

*The* ***Ethiopian Food and Drug Authority (EFDA)*** *is the sole national regulatory body responsible for enforcing WHO Good Manufacturing Practice (GMP) compliance within Ethiopia. Established under Proclamation No. 1112/2019, the EFDA ensures that every pharmaceutical facility—both domestic and international—adheres to stringent quality standards before its products can reach the Ethiopian market. Its enforcement toolkit is comprehensive, ranging from mandatory on-site and remote inspections to the "Market Authorization" process, which legally blocks any medicine not produced in a GMP-compliant factory. By conducting routine audits every few years and verifying "Corrective and Preventive Action" (CAPA) plans, the EFDA acts as the country’s primary gatekeeper for pharmaceutical quality and safety. Dear reviewers, these concepts are found in final paragraph of introduction, 4^th^ line.*

**4. State the penalties for non-compliance in Ethiopia.**

*Under Proclamation No. 1112/2019, the Ethiopian Food and Drug Authority (EFDA) enforces a tiered system of penalties designed to protect public health by ensuring pharmaceutical quality. Administrative penalties range from the immediate suspension or revocation of a facility’s GMP certificate and Marketing Authorization (MA) to mandatory product recalls and the physical sealing of non-compliant manufacturing sites. Beyond these regulatory actions, violators face severe criminal and financial penalties, including heavy fines and imprisonment for individuals involved in manufacturing falsified or substandard medicines. These measures ensure that quality standards remain a non-negotiable legal obligation, preventing the circulation of ineffective or dangerous drugs within the country. Dear reviewers, these concepts are found in final paragraph of introduction, 11^th^ line.*

**5. Define EFDA (Ethiopian FDA) and include it in the abbreviations section**

*The Ethiopian Food and Drug Authority (EFDA) is the federal regulatory body mandated by Proclamation No. 1263/2021 to protect public health by ensuring the safety, quality, and efficacy of medicines, food, medical devices, and cosmetics. Operating as an autonomous agency under the Ministry of Health, it serves as the nation's primary gatekeeper, overseeing the entire lifecycle of health products—from manufacturing inspections and product registration to post-market surveillance. By enforcing international standards like WHO-GMP and regulating the trade of alcohol and tobacco, the EFDA ensures that all health-related products circulating in the Ethiopian market meet strict scientific and legal requirements to prevent disease and disability. Dear reviewers, these concepts are found in final paragraph of introduction, 6^th^ line.*

**Updates on references**

- Following the reviewers' suggestions, we have added new information and references (#10 and #11). As a result, the bibliography has been updated and the reference numbering has changed.
- WHO References (#3): I updated the "Technical Report Series" (TRS) numbers. Reference #3 is specifically from TRS 1044, which is the landmark 2022 update for sterile manufacturing.

Thank you for your time.
