# Supplementary material for: Assessment of current good manufacturing practice (cGMP) compliance in pharmaceutical manufacturers in Ethiopia: Cross-sectional descriptive study
Source: PLoS One. 2026 Mar 9;21(3):e0343881. doi: 10.1371/journal.pone.0343881 (PMC12970858; doi:10.1371/journal.pone.0343881)
Supplement: Attachment — Submitted filename: Reviewer comments.docx [file pone.0343881.s004.docx]

This is a cross-sectional, facility-level assessment of cGMP implementation in six large-scale Ethiopian pharmaceutical manufacturers. The authors used a WHO-based checklist (S2) and scored sub-elements (FC=3, PC=2, NC=1) to produce company compliance percentages and identify weak areas (QA, QRM, HVAC, documentation, internal audit, complaint/recall). Strengthening local manufacturing quality systems is high priority worldwide; the paper addresses an important regulatory and public-health issue. Comparable regional analyses exist and make this work useful for national stakeholders.

However, few concerns have been raised for this paper. First, selection of only 6 companies, the sampling strategy risks selection bias, the manuscript does not describe how companies were recruited or whether non-participation occurred (response rate among 10 approached). This affects possible non-response bias, although I understand that companies might not be very cooperative sometimes. Second, The numerical conversion (FC=3, PC=2, NC=1) and the derived % thresholds ( >85%=high, 80–85%=medium, <80%=low) are not justified/validated. No sensitivity analysis or inter-rater reliability reported to show reproducibility between the two pharmacist assessors. Third, only 5 personnel per company (N=30) were interviewed, with such small cell counts the meaningfulness of some p-values is questionable and should be reported with caution. Fourth, no multivariable analysis to control confounding (e.g., company age, number of lines, certification status). Fifth, regarding data availability, authors claim “All relevant data are within the manuscript and Supporting Information” but raw checklists / per-company itemized scores are absent. For reproducibility, provide de-identified item-level datasets or an aggregated CSV in a public repository.

I recommend the authors to explain how the 6 companies were chosen from the 10 approached and discuss representativeness relative to the 12 large manufacturers in Ethiopia in a more detailed manner. Also, I advise the authors to report inter-rater reliability (Cohen’s kappa or % agreement) between the two pharmacists who collected/check-listed data. Provide the full checklist scoring rubric in the supplement (item-level scores per company, de-identified). Please add a methods paragraph justifying FC/PC/NC numeric mapping, explain why thresholds (>85%, 80–85%, <80%) were chosen (cite similar validation if available), or present sensitivity analyses showing how results shift with alternative cutoffs. Discuss selection bias, small sample size, potential Hawthorne effect (sites may temporarily improve practice during audit), lack of PAT adoption measurement detail, and language/translation limitations if any.

With the methodological clarifications and stronger transparency (itemized data, reliability stats) requested above, the manuscript could be acceptable.
